# Triboelectric nanogenerator (TENG) mass spectrometry of falsified antimalarials

**DOI:** 10.1002/rcm.8207

**Published:** 2018-08-13

**Authors:** Matthew C. Bernier, Anyin Li, Laura Winalski, Yunlong Zi, Yafeng Li, Céline Caillet, Paul Newton, Zhong Lin Wang, Facundo M. Fernández

**Affiliations:** ^1^ School of Chemistry and Biochemistry Georgia Institute of Technology Atlanta GA 30332 USA; ^2^ School of Materials Science and Engineering Georgia Institute of Technology Atlanta GA 30332 USA; ^3^ Lao‐Oxford‐Mahosot Hospital Wellcome Trust Research Unit (LOMWRU), Laos and Worldwide Antimalarial Resistance Network, Centre for Tropical Medicine & Global Health University of Oxford Oxford UK; ^4^ Beijing Institute of Nanoenergy and Nanosystems Chinese Academy of Sciences, National Center for Nanoscience and Technology (NCNST) Beijing 100083 China; ^5^ Institute of Bioengineering and Biosciences Georgia Institute of Technology Atlanta GA 30332 USA

## Abstract

**Rationale:**

An epidemic of low‐quality medicines continues to endanger patients worldwide. Detection of such ‘medicines’ requires low cost, ambient ionization sources coupled to fieldable mass spectrometers for optimum sensitivity and specificity. With the use of triboelectric nanogenerators (TENGs), the charge required to produce gas‐phase ions for mass analysis can be obtained without the need for high‐voltage electrical circuitry, simplifying and lowering the cost of next‐generation mass spectrometry instruments.

**Methods:**

A sliding freestanding (SF) TENG was coupled to a toothpick electrospray setup for the purposes of testing if falsified medicines could be fingerprinted by this approach. Extracts from both genuine and falsified medicines were deposited on the toothpick and the SF TENG actuated to generate electrical charges, resulting in gas‐phase ions for both active pharmaceutical ingredients and excipients.

**Results:**

Our previous work had shown that direct analysis in real time (DART) ambient mass spectrometry can identify the components of multiple classes of falsified antimalarial medicines. Experiments performed in this study show that a simple extraction into methanol along with the use of a SF TENG‐powered toothpick electrospray can provide similar detection capabilities, but with much simpler and rugged instrumentation, and without the need for compressed gases or high‐voltage ion source power supplies.

**Conclusions:**

TENG toothpick MS allows for rapid analyte ion detection in a safe and low‐cost manner, providing robust sampling and ionization capabilities.

## 1 INTRODUCTION

Poor Quality Medicines (PQM) are a major global health concern of increasing significance. They not only constitute an impediment for patients to receive the expected treatment, but may also be a key but neglected driver of antimicrobial resistance.^1^ Lack of effective treatment, decreased public confidence, significant financial damage, and adverse reactions are only some of the known effects of the worldwide prevalence of PQM.^2^

Three main categories of PQM exist: falsified (produced by criminals), substandard (due to negligent errors in manufacturing), and degraded (arising from inadequate storage through the distribution chains).^3^ The term ‘counterfeit’, very commonly used to describe PQM, is not adequate to describe falsified medicines and is only preferred when describing trademark infringement.^4^ PQM have been found in both low‐ and middle‐income countries (L/MICs), as well as in wealthier countries with well‐functioning medicine regulatory agencies.^5^ Despite ongoing efforts and interventions worldwide, falsification of medicines remains a lucrative and prevalent activity.^6^

Pharmaceutical forensics can significantly gain from advances in portable analytical instrumentation for field use: detection of PQM at point of sale, clinics, or pharmacies could effectively prevent patients being exposed to these ‘products’. Chemical analysis techniques typically used for PQM field detection include Raman spectroscopy, NIR spectroscopy, thin‐layer chromatography, colorimetry, dissolution assays, paper analytical devices, and others.^7^ However, field implementation of these techniques usually forces a compromise between cost and performance, necessarily reducing sensitivity and specificity. For this reason, field detection and forensic characterization of PQM typically rely on the use of simpler field techniques for screening purposes, in combination with tiered laboratory approaches.^8^ Mass spectrometry (MS), once a complex technique available only in centralized facilities, has progressively shown better performance in field applications through portable, many times handheld, mass spectrometers,^9^ achieving the right balance between resolution, sensitivity, power consumption and cost. The performance of such mass spectrometers is now sufficient to meet the challenges posed by pharmaceutical forensics, which include the detection of falsified medicines in pass/fail scenarios, the identification of ‘wrong active ingredients’ present in fakes, and semi‐quantitation of the correct active pharmaceutical ingredients (APIs) to distinguish high‐quality samples from degraded and substandards, the latter sometimes being more prevalent than falsified medicines.^10^, ^11^, ^12^

In order to take advantage of advances in portable mass spectrometers, a necessary condition is the ability to sample and ionize neutral analytes with minimal sample preparation^13^ as well as simple high‐voltage supply apparatus. Here, we present the first application of the recently developed TENG MS ion source^14^ to pharmaceutical forensics using a dry wooden toothpick to sustain electrospray ionization (ESI).^15^ Samples of both genuine and falsified antimalarial tablets were subjected to a quick extraction in methanol, and a small volume of the extract was deposited on the toothpick tip. Manual (human‐powered) actuation of a SF TENG connected to the toothpick readily led to mass spectra that provided reproducible, qualitative information that was comparable with that obtained from conventional and resource‐intensive ionization approaches, such as direct analysis in real time (DART).^16^

## 2 EXPERIMENTAL

### 2.1 Materials, supplies, and sample preparation

World Health Organization (WHO) pre‐qualified artemether‐lumefantrine tablets (10 mg/tablet co‐formulated with 120 mg/tablet, respectively) were obtained directly from a reputable provider and were used as genuine comparators. ACT samples collected in parts of Sub‐Saharan Africa during active law‐enforcement operations were used to test the TENG‐MS approach. These samples were suspected of being falsified through initial Raman spectroscopy screening. Follow‐up fingerprinting studies on these samples through DART‐MS experiments confirmed the absence of the expected APIs, and in some cases the presence of wrong APIs.^16^

Solvents used for tablet extraction included Nanopure water (Barnstead Diamond; Thermo Fisher Scientific, Waltham, MA, USA) and HPLC grade methanol (99.9%; Sigma‐Aldrich, St Louis, MO, USA). Tablet extraction was performed by adding approximately 1 mg of crushed tablet powder to 1 mL of methanol in a plastic vial, vortexing of the resulting suspension, and allowing it to equilibrate for 8 h. This extraction procedure was selected so as to allow qualitative comparisons with previous ESI‐MS work,^16^ but it should be noted that shorter extraction times could also viable for detection of the main tablet components.

Calibration of the mass spectrometer was performed with a solution of sodium formate in water (20 μM) pipetted onto the wooden tip electrospray immediately prior to the analytical runs. Glucose, mannitol, chloramphenicol, and ciprofloxacin standards were purchased from Sigma‐Aldrich and used to confirm chemical assignments for wrong active ingredients found in falsified antimalarials.

### 2.2 TENG wooden‐tip MS analysis of antimalarial tablets

**Figure 1:**
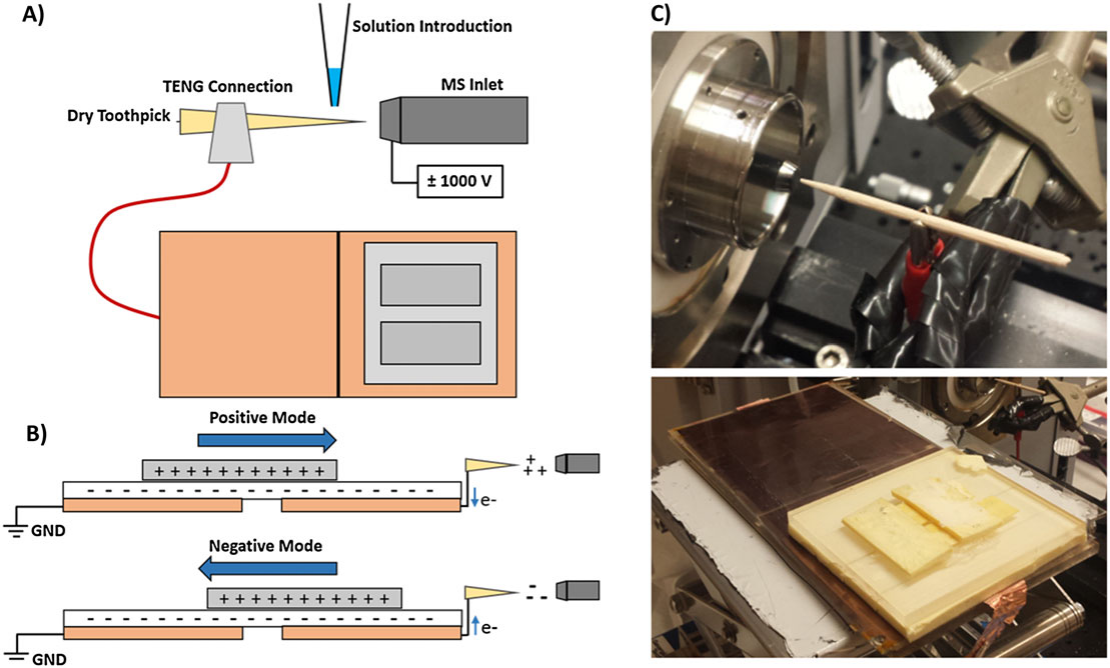
(A) Schematic of the sliding freestanding TENG wooden‐tip setup used in these experiments. (B) The two TENG copper electrodes on the bottom layer are shown in light orange, while the sliding piece with lower charge density is shown in gray and the insulating middle layer is shown in white. Charge is produced by the SF TENG in discrete pulses stemming from the flow of charge created when the top electrode is moved from one end to the other end of the stationary electrode. This charge is directed to the wooden tip facing the inlet. (V) Close‐up image of the dry toothpick and mass spectrometer inlet setup as well as the SF TENG source used [Color figure can be viewed at http://wileyonlinelibrary.com]

Dry wooden toothpicks supplied from Haioreum (South Korea) were initially 8 cm in length, 3 mm in diameter, and cut to 4 cm to enable placement onto a clip connected directly to the SF TENG power source. The toothpick was not altered in any other way. Figure 1 depicts the TENG wooden‐tip MS setup. The tip was placed approximately 1 cm away from the inlet to the mass spectrometer. Best results were observed when the alligator clip supplying the charge was placed ~0.5 cm from the toothpick tip so as to minimize the length of dry toothpick wood present between the deposited extraction solution and the electrical contact, minimizing overall electrical resistance.

A hybrid high‐resolution quadrupole time‐of‐flight (QTOF) mass spectrometer (MicrOTOF‐Q1; Bruker, Bremen, Germany) was used for initial experiments, while reproducibility experiments (see supporting information) were performed on a Synapt G2‐S QTOF from Waters (Wilmslow, UK). This instrument was operated in both positive and negative ion mode as needed, in the *m/z* 50–1000 range. To ensure ion transmission, and due to the reverse polarity scheme employed in Bruker ion sources, a ± 1000 V capillary bias was applied in all cases. The endplate offset was set to +500 V in positive and − 500 V in negative mode, with a 5.0 eV ion energy offset, 2.7 mbar fore pressure, and a TOF pressure of 4.42 × 10^−7^ mbar. For ESI experiments, the QTOF instrument was used with the following settings: +4500 V capillary voltage, −500 V end plate offset, 2.0 L min^−1^ flow rate and temperature of 200°C for the N_2_ drying gas, 1.0 bar pressure for the N_2_ nebulizing gas, and fore and TOF vacuum pressures of 2.41 mbar and 1.09 × 10^−7^ mbar, respectively.

With the mass spectrometer acquiring continuously, 2 μL of methanolic sample extract were pipette‐deposited directly onto the wooden tip. Following sample deposition, the top piece of the SF TENG device was manually moved from the grounded side of the TENG assembly towards the side connected to the wooden tip, producing positive ions (Figure 1B). This motion produced a transient signal in the total ion chronogram that was later averaged over its whole time span. Typically, between one and three ionization events were possible from a single 2 μL methanol sample spotted on the tip before the sample was either consumed or absorbed into the tip surface. Additional coating to induce higher efficiency of the TENG tip spray may improve signal and the number of possible sampling events. Negative ion mode runs were performed by sliding the TENG electrode from the wooden tip side to the grounded side. The static part of the SF TENG consisted of two 75 × 60 mm^2^ rectangles of Cu film deposited onto fluorinated ethylene propylene (FEP) film, separated by a 75 × 1 mm^2^ uncoated rectangular section, mounted on an acrylic support. The movable SF TENG part was made of Cu foil (55 × 65 mm^2^), also mounted onto an acrylic board. To operate the TENG, the movable part was placed on top of the static portion, which was fixed onto a stage placed underneath the ion source. Both parts were oriented so that the two metal layers were separated by the FEP layer (Figure 1). The movable part was slid between the two positions corresponding to the two Cu‐coated regions of the static part, with a travel distance of 60 mm.

### 2.3 DART analysis of antimalarial tablets

Analysis of the solid ACT tablets was performed with a commercial DART‐SVP ion source (IonSense Inc., Saugus, MA, USA), directly sampling powder from crushed antimalarial tablets, as previously described.^16^ Ultra‐grade 99.999% pure helium gas (Airgas, Atlanta, GA, USA) flowing at 2.2 L min^−1^ was used to generate the metastable helium plasma. A grid electrode voltage of +250 V was used, with a gas temperature of 500°C. A gas‐ion separator tube (GIST) provided additional pumping of the heated helium gas preventing it from overloading the vacuum system of the mass spectrometer. The DART source was placed 1 cm away from the GIST opening. Tablet powder was introduced between the GIST and the DART nozzle using a borosilicate glass capillary, and held in place for 5 s to generate DART‐MS spectra.

For both DART and wooden‐tip ESI spectra, background subtraction was carried out as both the borosilicate glass capillary and the wooden tips used for each method generated their own sets of background ions. For DART, a clean glass capillary was introduced into the source in front of the mass spectrometer inlet for the same amount of time and in the same position as the tablet. For TENG wooden‐tip MS, 2 μL of pure methanol were spotted onto the tip in the same manner as for the extracted sample solution. This ‘pre‐conditioning’ of the wooden tip also enhanced ionization, and was thus performed prior to the analysis of all samples. Background spectra are provided in Figures S1 and S2 (supporting information).

## 3 RESULTS AND DISCUSSION

### 3.1 TENG wooden‐tip MS and DART‐MS of genuine antimalarials

**Figure 2:**
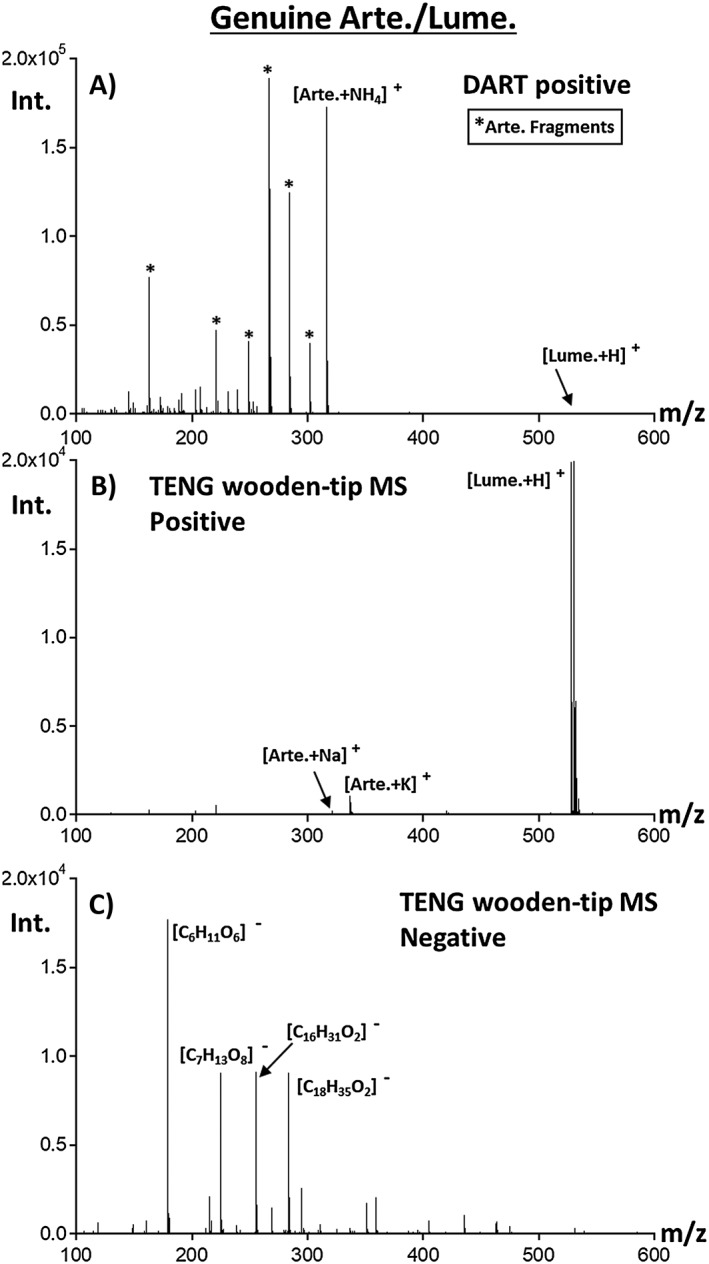
Comparison of SF TENG wooden‐tip MS and DART‐MS results for the analysis of genuine artemether‐lumefantrine antimalarial tablets. For TENG wooden‐tip MS, powdered samples were extracted in methanol and the extract was deposited on the wooden tip. (A) DART positive mode MS analysis. (B) and (C) show TENG wooden‐tip MS results in positive and negative ion mode, respectively

A genuine artemether‐lumefantrine ACT was tested to determine the ability of TENG wooden‐tip MS to generate positive or negative ions (Figures 2B and 2C). Experiments were also carried out with a DART ion source in positive ion mode using exactly the same mass spectrometer for comparison purposes (Figure 2A). DART ionization produced signals for both the APIs, artemether and lumefantrine. For artemether, the ammoniated precursor ion at *m/z* 316.16, along with a number of fragment ions (Figure 2A, labeled with *) that included [M − CH_2_ + NH_4_]^+^ at *m/z* 302.20, [M − CH_3_OH + NH_4_]^+^ at *m/z* 284.19, [M − CH_3_OH + H]^+^ at *m/z* 267.16, [M − CH_3_OH − H_2_O + H]^+^ at *m/z* 249.15, [M − CH_3_OH − CO − H_2_O + H]^+^ at *m/z* 221.15, and [M − CH_3_OH − C_4_H_8_O_3_ + H]^+^ at *m/z* 163.11, were detected. Lumefantrine was observed as the protonated precursor ion at *m/z* 528.16 with no fragment ions present. The abundance of the lumefantrine ionic species was much lower than for artemether species, at approximately 1% relative intensity. This effect was associated with the lower volatility of this heavier species, leading to lower ion generation efficiency due to the thermal desorption step involved in DART.

Figure 2B shows the results for the TENG wooden‐tip MS analysis of the same genuine tablet in positive ion mode, with a much higher proportion of the protonated lumefantrine molecule at *m/z* 528.16, together with artemether being detected as both the [M + Na]^+^ ion at *m/z* 321.17 and the [M + K]^+^ ion at *m/z* 337.14. Low‐abundance signals for some of the artemether fragment ions, including [M − CH_3_OH − C_4_H_8_O_3_ + H]^+^ at *m/z* 163.11 and [M − CH_3_OH − CO − H_2_O + H]^+^ at *m/z* 221.15, were also observed. The TENG wooden‐tip MS results in negative ion mode showed no ions for the APIs, a fact to be expected as both ingredients cannot be easily deprotonated. Instead, signals for deprotonated stearic acid [C_18_H_35_O_2_]^−^ at *m/z* 283.26 and palmitic acid [C_16_H_31_O_2_]^−^ at *m/z* 255.23, two of the excipients, were detected. Additional species at *m/z* 179.06 representing a deprotonated hexose ([C_6_H_11_O_6_]^−^) and an unknown ion at *m/z* 225.06 ([C_7_H_13_O_8_]^−^) were observed.

It was also observed that with a single sliding movement of the TENG top electrode, the overall ion abundance was comparable with that seen by sampling ~1 mg of crushed solid tablet by DART for ~ 5 s. The higher abundance of lumefantrine observed by TENG‐MS was also observed in our previous experiments using ESI.^16^ The opposite was true for artemether, and this is probably because this compound has no easily protonated basic site unlike the tertiary amine on lumefantrine. An observed advantage of TENG was the ability to form sodiated and potassiated artemether adduct ions without the excessive fragmentation seen in DART, while also avoiding the need for compressed gases.

### 3.2 Distinguishing falsified ACT antimalarials with TENG wooden‐tip MS

**Figure 3:**
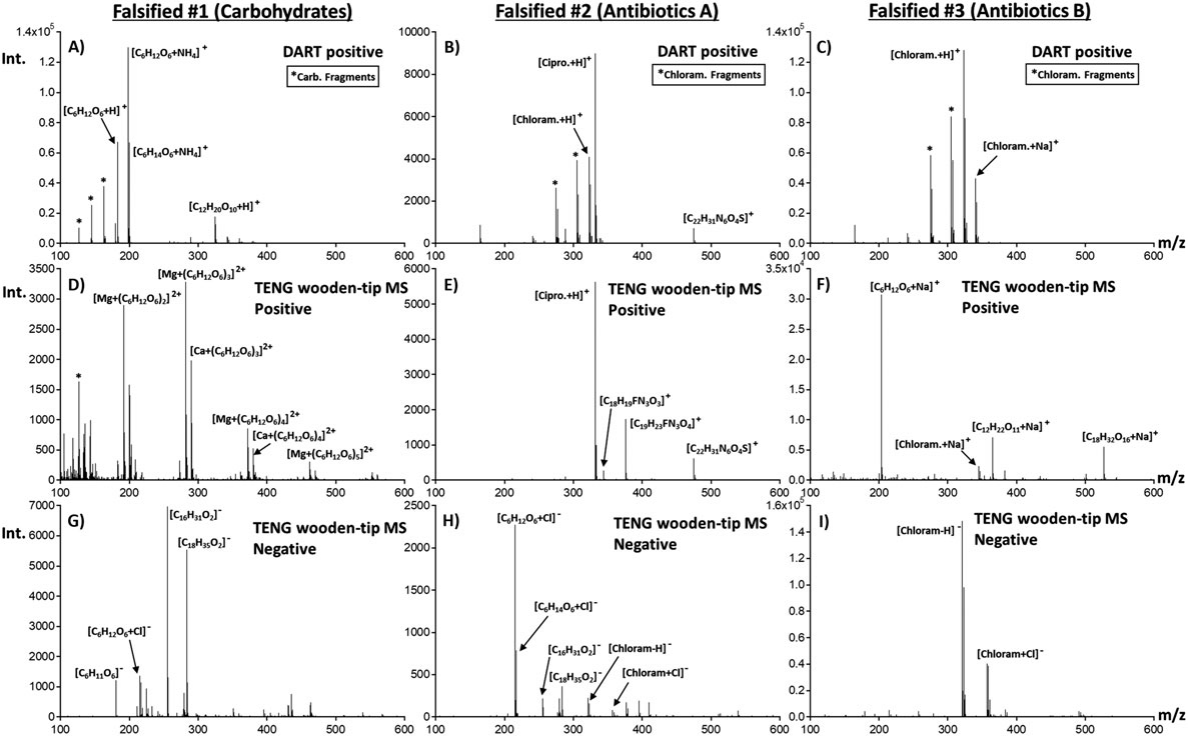
Comparison of TENG wooden‐tip MS and DART‐MS results for the analysis of three different classes of falsified artemether‐lumefantrine antimalarial tablets: (A–C) DART positive mode; (D–F) SF TENG positive mode; and (G–I) negative mode TENG wooden‐tip MS

Figure 3 shows results for the TENG‐MS analysis of three common types of falsified ACT antimalarials collected in Sub‐Saharan Africa. These three types, previously fingerprinted by DART,^16^ included: ‘Falsified #1’ containing no active ingredients but rather a set of sugars with elemental formulae C_6_H_12_O_6_ and C_6_H_14_O_6_, along with sucrose and maltitol; ‘Falsified #2’ which contained a mixture of two antibiotics, chloramphenicol and ciprofloxacin; and ‘Falsified #3’ only containing the antibiotic ciprofloxacin. Also shown in Figures 3A–3C are the DART positive mode spectra for comparison purposes.

For falsified ACTs of type #1, various carbohydrates were detected, with abundances differing between DART and TENG wooden‐tip MS results. The ammoniated and protonated forms of glucose at *m/z* 198.10 and 181.07 ([C_6_H_12_O_6_ + NH_4_]^+^ and [C_6_H_12_O_6_ + H]^+^) as well as mannitol species such as [C_6_H_14_O_6_ + NH_4_]^+^ and [C_6_H_14_O_6_ + H]^+^ along with their water loss fragment ions, and other carbohydrates at *m/z* 325.11 ([C_12_H_20_O_10_ + H]^+^), were the predominant species detected by DART‐MS (Figure 3A). A positive mode TENG mass spectrum for this type of sample is shown in Figure 3D, indicating Mg^2+^ and Ca^2+^ adduct formation with C_6_H_12_O_6_ repeating units, so that the spectrum was dominated by [Mg + (C_6_H_12_O_6_)_n_]^2+^ and [Ca + (C_6_H_12_O_6_)_n_]^2+^ species. This series of carbohydrate‐metal adducts were detected up to *n* = 6 at *m/z* 552.18 for [Mg + (C_6_H_12_O_6_)_6_]^2+^ and *m/z* 560.17 for [Ca + (C_6_H_12_O_6_)_6_]^2+^, providing a unique fingerprint. In addition, negative ion mode TENG wooden‐tip MS showed intense peaks from stearic and palmitic acids (Figure 3G), suggesting that magnesium and calcium stearate were probably the excipients used. The results shown in Figure 3G also indicate the presence of the glucose chloride adduct ion [C_6_H_12_O_6_ + Cl]^−^ at *m/z* 215.03, and deprotonated glucose ([C_6_H_11_O_6_]^−^). The stearic and palmitic acid signals were the most intense, however, which was different from the genuine sample where the base peak was that of deprotonated glucose.

Falsified samples of type #2 were found to contain a mixture of the antibiotics chloramphenicol and ciprofloxacin following initial analysis with DART‐MS (Figure 3B). Signals corresponding to protonated ciprofloxacin [C_17_H_18_FN_3_O_3_ + H]^+^ at *m/z* 332.14, protonated chloramphenicol [C_11_H_12_Cl_2_N_2_O_5_ + H]^+^at *m/z* 323.02, and also a significant signal at *m/z* 475.21 with elemental formula [C_22_H_31_N_6_O_4_S]^+^, probably sildenafil, were detected. Ciprofloxacin and sildenafil were also the major species detected by positive mode TENG wooden‐tip MS, as shown in Figure 3E, with relative abundances similar to what was observed by DART. No ions for chloramphenicol were detected, this observation being consistent with previous observations during ESI analysis.^16^ Additional species at *m/z* 344.15 and 376.17 were observed. The ion at *m/z* 376.17 suggests the presence of protonated gatifloxacin [C_19_H_22_FN_3_O_4_ + H]^+^, an antibiotic from the fluoroquinolone family that was only detected with TENG wooden‐tip MS, requiring further investigations into the identity of this species. Results for negative ion mode TENG wooden‐tip MS presented in Figure 3H showed similarities with the genuine sample (Figure 2C) and the type #1 fake (Figure 3G) in terms of signals for palmitic and stearic acid along with the chloride adduct ion of glucose [C_6_H_12_O_6_ + Cl]^−^. Clear signals for two chloramphenicol species, [C_11_H_11_Cl_2_N_2_O_5_]^−^ and [C_11_H_12_Cl_2_N_2_O_5_ + Cl]^−^ were also observed, suggesting that the absence of chloramphenicol signals in Figure 3E is probably due to ion suppression and not to limited solubility in the extraction solution. Clearly, avenues for mitigating this type of effect are needed to achieve the full potential of TENG wooden‐tip MS for forensic purposes.

Finally, a type #3 falsified ACT sample was investigated using both DART‐ and TENG‐MS. Similar to what was observed for type #2 samples, type #3 samples showed chloramphenicol species at *m/z* 323.02 ([C_11_H_12_Cl_2_N_2_O_5_ + H]^+^), as well as [C_11_H_12_Cl_2_N_2_O_5_ + Na]^+^ at *m/z* 345.00 by DART‐MS (Figure 3C). Much lower abundance chloramphenicol ion signals were detected by TENG wooden‐tip MS following extraction (Figure 3F), and higher abundance carbohydrate signals. The most intense chloramphenicol signal in the spectrum was at *m/z* 345.00 from the sodium adduct ([C_11_H_12_Cl_2_N_2_O_5_ + Na]^+^). The dominant peak in positive ion mode was at *m/z* 203.05, corresponding to sodiated glucose [C_6_H_12_O_6_ + Na]^+^. The second and third species in terms of abundance were the sodiated disaccharide [C_12_H_22_O_11_ + Na]^+^ at *m/z* 365.10 and the sodiated trisaccharide [C_18_H_32_O_16_ + Na]^+^ at *m/z* 527.16. While not shown, this sodiated carbohydrate series continued up to the pentasaccharide at *m/z* 851.25 with progressively lower abundances for the longer chains. This series of polymeric species suggests the presence of starch in the falsified tablet, a common and inexpensive pharmaceutical excipient.

Finally, the negative ion mode mass spectrum for a type #3 falsified ACT (Figure 3I) revealed a major set of species related to chloramphenicol, indicating that, unlike type #2 falsified samples that were richer in sugars, this type of sample contained higher concentration of this wrong active ingredient. Clearly, TENG wooden‐tip MS enabled fingerprinting this type of poor quality pharmaceutical successfully, providing information to source its origins based on chemical composition, and providing a tool for finding chemical similarities with other falsified samples that might be collected in other locations, but produced in a common source. Despite using manual TENG actuation, the spectra collected were highly reproducible, as shown in Figure S3 (supporting information) using a chloramphenicol‐containing tablet. This reproducibility makes qualitative comparisons simpler without the need for complex equipment that would void the simplicity of the TENG ion source.

## 4 CONCLUSIONS

The triboelectric nanogenerator wooden‐tip MS method offer a simple, affordable way to generate ions, producing information that is comparable with, or even richer than, that produced with ion sources such as DART. Even if manually actuated, this new type of triboelectric ion source yields satisfactory results in terms of producing chemical fingerprints of falsified medicines lacking the expected active ingredient, with the additional advantage of allowing identification of any ‘wrong’ active ingredients that might be present. It is expected that, when coupled to fieldable or miniature mass spectrometers, triboelectric ion sources will lead to a new generation of less costly instrument platforms that can be used for routine medicine quality monitoring in the field and the clinic. Although positive and negative ion mode were tested independently for TENG wooden‐tip MS in the experiments described here, the ability of triboelectric sliding freestanding devices to generate charges of opposite polarity in any given actuation cycle could be further leveraged to improve analytical performance. Along these lines, experiments with TENG and rapid MS polarity switching modes should further increase sample throughput, yielding information about both active ingredients and excipients in a single experiment.

## Supporting information


**Supplemental Figure 1:** Positive ion mode background spectra generated via TENG wooden‐tip MS with three separate toothpick tips.
**Supplemental Figure 2:** Negative ion mode background spectra generated via TENG wooden‐tip MS with three separate toothpick tips.
**Supplemental Figure 3:** Negative ion mode spectra generated via TENG wooden‐tip MS for methanol‐extracted chloramphenicol‐containing tablets with three separate toothpick tips.
