# Supplementary material for: Triboelectric nanogenerator (TENG) mass spectrometry of falsified antimalarials
Source: Rapid Commun Mass Spectrom. 2018 Aug 13;32(18):1585–90. doi: 10.1002/rcm.8207 (PMC6120538; doi:10.1002/rcm.8207)
Supplement: Supplementary file 1 — Supplemental Figure 1: Positive ion mode background spectra generated via TENG wooden‐tip MS with three separate toothpick tips. Supplemental Figure 2: Negative ion mode background spectra generated via TENG wooden‐tip MS with three separate toothpick tips. Supplemental Figure 3: Negative ion mode spectra generated via TENG wooden‐tip MS for methanol‐extracted chloramphenicol‐containing tablets with three separate toothpick tips. [file RCM-32-1585-s001.docx]

Supplementary Information for “Triboelectric Nanogenerator (TENG) Mass Spectrometry of Falsified Antimalarials” by Bernier *et al.*

**
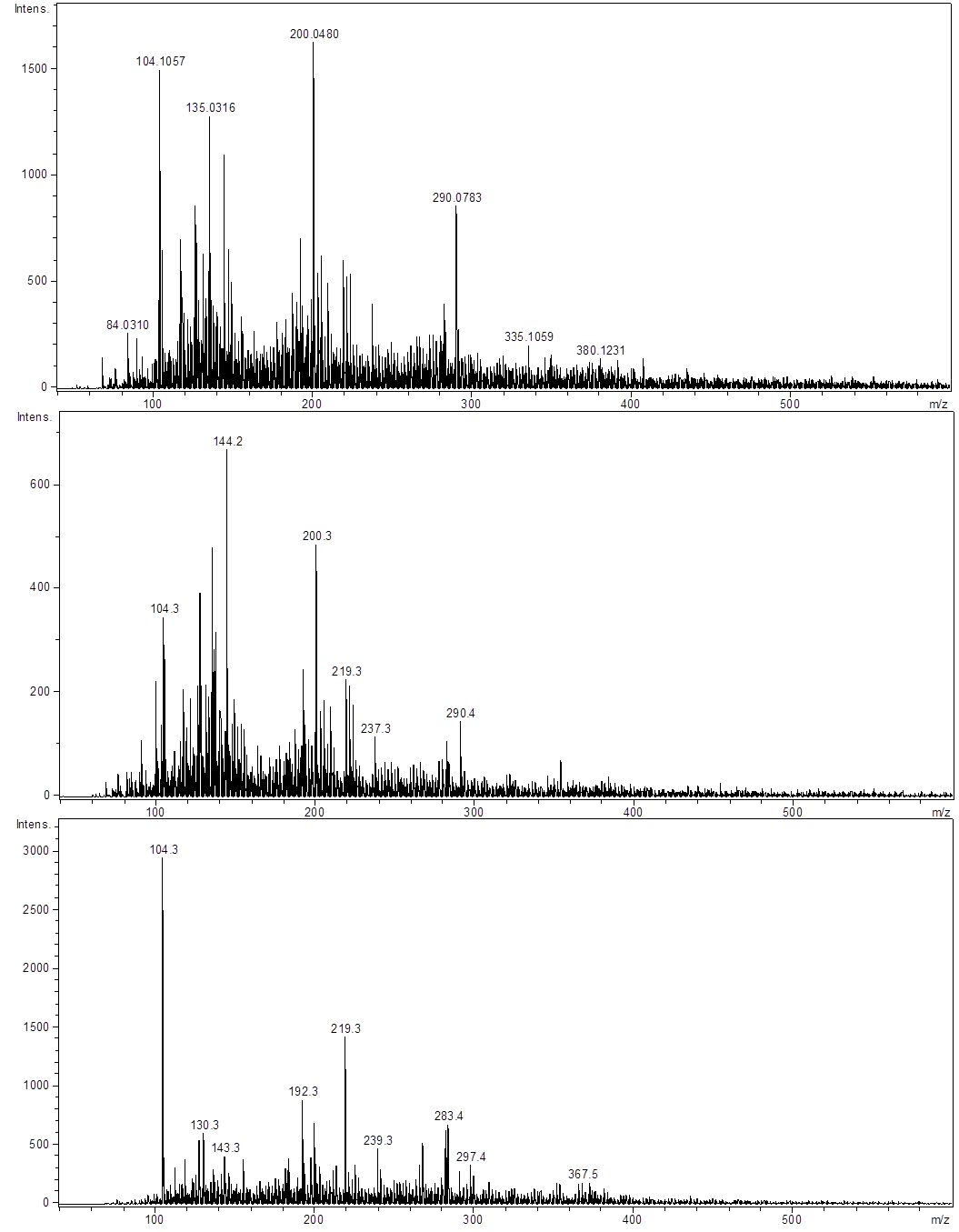
**

**Supplemental Figure 1:** Positive ion mode background spectra generated via TENG wooden-tip MS with three separate toothpick tips.

**
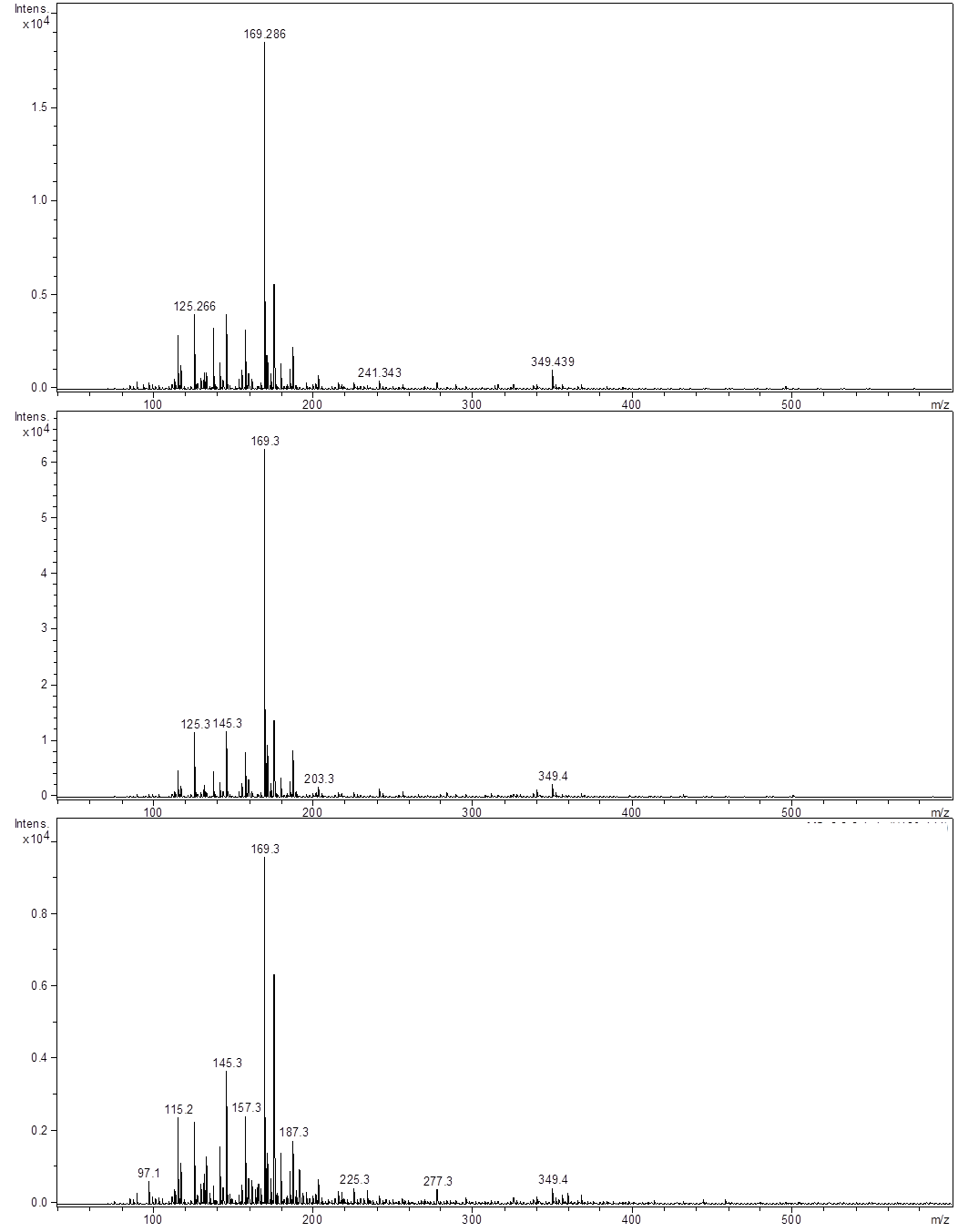
**

**Supplemental Figure 2:** Negative ion mode background spectra generated via TENG wooden-tip MS with three separate toothpick tips.

**
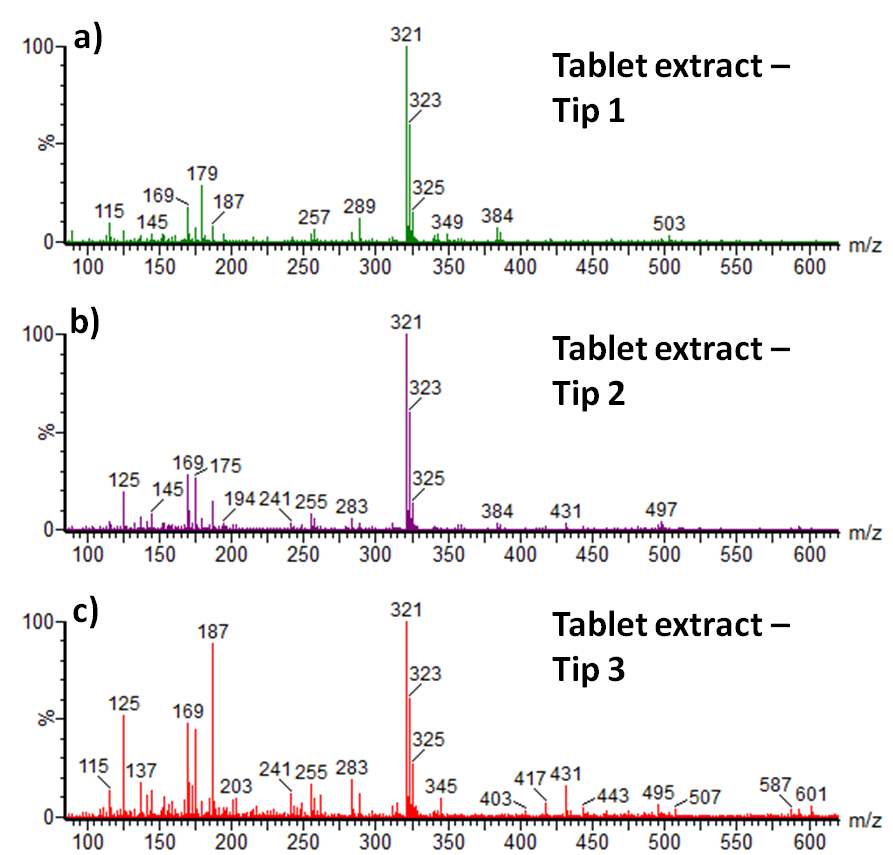
**

**Supplemental Figure 3:** Negative ion mode spectra generated via TENG wooden-tip MS for methanol-extracted chloramphenicol-containing tablets with three separate toothpick tips.
